# Automatic Vertebral Rotation Angle Measurement of 3D Vertebrae Based on an Improved Transformer Network

**DOI:** 10.3390/e26020097

**Published:** 2024-01-23

**Authors:** Xing Huo, Hao Li, Kun Shao

**Affiliations:** 1 School of Mathematics, Hefei University of Technology, Hefei 230601, China; huoxing@hfut.edu.cn (X.H.); 2021111431@mail.hfut.edu.cn (H.L.); 2 School of Software, Hefei University of Technology, Hefei 230601, China

**Keywords:** predictive models, deep learning, attention works, point cloud, automatic measurement

## Abstract

The measurement of vertebral rotation angles serves as a crucial parameter in spinal assessments, particularly in understanding conditions such as idiopathic scoliosis. Historically, these angles were calculated from 2D CT images. However, such 2D techniques fail to comprehensively capture the intricate three-dimensional deformities inherent in spinal curvatures. To overcome the limitations of manual measurements and 2D imaging, we introduce an entirely automated approach for quantifying vertebral rotation angles using a three-dimensional vertebral model. Our method involves refining a point cloud segmentation network based on a transformer architecture. This enhanced network segments the three-dimensional vertebral point cloud, allowing for accurate measurement of vertebral rotation angles. In contrast to conventional network methodologies, our approach exhibits notable improvements in segmenting vertebral datasets. To validate our approach, we compare our automated measurements with angles derived from prevalent manual labeling techniques. The analysis, conducted through Bland–Altman plots and the corresponding intraclass correlation coefficient results, indicates significant agreement between our automated measurement method and manual measurements. The observed high intraclass correlation coefficients (ranging from 0.980 to 0.993) further underscore the reliability of our automated measurement process. Consequently, our proposed method demonstrates substantial potential for clinical applications, showcasing its capacity to provide accurate and efficient vertebral rotation angle measurements.

## 1. Introduction

Adolescent idiopathic scoliosis, characterized by complex three-dimensional deformities involving coronal, sagittal, and axial imbalances, demands precise measurement for comprehensive evaluation. Accurate assessment is crucial for gauging scoliosis severity, choosing the most appropriate treatment strategies, and effectively monitoring disease progression. Notably, vertebral rotation plays a pivotal role in the enigmatic pathogenesis of scoliosis [1]. Vrtovec [2] introduced a quantitative method automating the identification of the vertebral body’s median plane, enhancing rotation evaluation. Wang [3] validated a novel three-dimensional ultrasound technique for quantifying vertebral rotation, highlighting its practicality. Recently, the orthopedic community has increased its focus on axial plane rotational deformities, reflecting heightened interest in this aspect [4,5]. This collective research underscores the growing importance of accurately measuring vertebral rotation, enhancing our understanding of scoliosis etiology and clinical management.

In cases of spinal curvature, CT images may obscure parts of vertebral bodies and pedicles, necessitating observations from multiple angles. Identifying individual pedicles on a curved spine using 2D images can be challenging [6,7]. However, a 3D vertebral model offers a promising solution, reducing potential CT imaging errors and providing a better understanding of spinal structures. Utilizing spatial angle assessments on these models can yield highly precise scoliosis angle measurements, which is crucial for guiding scoliosis treatment.

Previous methods relied on spinous process positions or pedicle shadows in 2D images, limiting angle estimations. In contrast, our proposed technique directly employs a 3D vertebral model, accurately establishing a vertebra’s local coordinate system by incorporating data from end plates and pedicle positions. This innovation can enhance angle measurements and improve the precision of spinal assessments, with the primary aim of reducing errors resulting from vertebral asymmetry, leading to more reliable and accurate results.

The key contributions of our work are as follows:

(1) We propose a novel clinically relevant method for measuring vertebral rotation angles. Addressing the limitations of traditional two-dimensional imaging techniques, we introduce an automated angle measurement approach specifically designed for three-dimensional vertebral models. This represents a significant advancement over traditional two-dimensional imaging techniques;
(2) We propose a transformer-based point cloud segmentation network that incorporates distance distribution entropy into the corresponding point cloud downsampling algorithm. It is noteworthy that our approach achieves significant progress in predicting the upper and lower endplates of specific vertebrae.

## 2. Related Work

### 2.1. Point Cloud Processing

Convolutional neural networks (CNNs) have significantly expanded their role in recognizing and segmenting geometric data, achieving breakthroughs in image classification [8]. However, 3D point cloud data lacks the standardized alignment principles of images due to its direct embedding in three-dimensional space.

A pioneering step in this direction was taken by Charles R. Qi et al., who introduced the PointNet network architecture, later refined as PointNet++ [9,10]. PointNet was the first deep neural network capable of directly processing raw point clouds, performing tasks such as point cloud classification and semantic segmentation using inherent point cloud data. Subsequently, researchers addressed PointNet’s limitations and developed methodologies for directly utilizing point cloud data in various point cloud analysis applications [11], emphasizing the interrelationships between points within the PointNet++ framework. Mao et al. [12] have proposed a new interpolation convolution operation, InterpConv, to address the learning and understanding challenges of point cloud features. To address the challenge of Multilayer Perceptrons (MLPs) inadequately capturing the geometric structure and contextual information of point clouds, Xie et al. [13] proposed GRNet. They regularized unordered point clouds into a 3D grid, ultimately achieving improved performance in point cloud completion tasks.

Recent years have seen the integration of convolution into point cloud data for point cloud segmentation [14,15,16,17]. Attention mechanisms [18], particularly self-attention mechanisms, have proven effective in capturing contextual information, making them suitable for point cloud processing tasks. The emergence of Point Transformer (PT) [19] and Point Cloud Transformer (PCT) [20] represents successful models for point cloud segmentation leveraging the transformer mechanism.

### 2.2. Vertebral Rotation Angle Measurement

The advent of deep learning technology has brought forth a novel wave of interdisciplinary applications across various engineering domains. In the realm of vertebral rotation angle measurement, neural network-based methodologies have garnered significant attention for automating the segmentation of vertebral bodies and pedicles. Noteworthy contributions include the work of Bakhous et al. [21], who introduced a CNN-based regression model aimed at enhancing vertebral pedicle localization and the estimation of vertebral rotation angles. Veena Logithasan et al. [22] developed a CNN-powered machine learning algorithm to autonomously compute the AVR (Apical Vertebral Rotation) on PAx radiographs using the Stokes method. Shahin Ebrahimi et al. [23] devised an automated pedicle detection system, employing image analysis, machine learning, and rapid manual landmark identification, serving as a quantitative VAR (Vertebral Apical Rotation) assessment tool for scoliosis patients. Zhang et al. [24] pioneered a computer-aided approach, integrating Hough transform and snake model techniques to semi-automatically gauge the Cobb angle and vertebral rotation on PAx radiographs. Devlin G. et al. [25] conducted spinal parameter measurements and correlation evaluations using standing PA radiographs, employing the Stokes method for vertebral rotation angle quantification. Quang N. Vo et al. [26] explored the reliability and accuracy of AVR measurements utilizing centerpoints from the vertebral body or transverse process on three-dimensional ultrasound images. Daniel Forsberg et al. [27] devised a fully automated approach for estimating AVR through images from computed tomography scans. An examination of recent research into vertebral rotation angle measurement methods reveals that a predominant focus remains on automated measurements derived from 2D CT images. However, these 2D approaches are susceptible to projection bias and often overlook the patient’s transverse plane. These conventional 2D techniques fail to fully capture the intricacies of three-dimensional spinal curvature deformities, particularly in the context of vertebral axial rotation, which holds considerable significance.

### 2.3. EOS Imaging System

The EOS imaging system is a cutting-edge, low-radiation, and highly accurate method for assessing spinal curvature. Some studies [28,29,30,31] have used EOS images to create comprehensive 3D spine reconstructions, forming the basis for measuring vertebral rotation angles with specialized software. While EOS imaging has been used to reconstruct 3D vertebral models in certain studies, measuring vertebral rotation angles often involves time-consuming manual point marking. In contrast, our method allows for direct and automated determination of these angles from the 3D models. Importantly, our automated approach shows strong agreement with manual measurements, enhancing its suitability for clinical assessments.

## 3. Materials and Methods

### 3.1. Point Cloud Sampling Method

In the context of point cloud downsampling, selecting a subset of original points to represent the entire point cloud proves effective in reducing the overall number of points, thereby minimizing storage and processing costs. Common downsampling methods encompass random sampling, voxel downsampling, grid downsampling, adaptive sampling, and other techniques.

To attain a uniformly distributed point cloud, we opt for the Farthest Point Sampling (FPS) algorithm. However, given the uneven density in vertebral point cloud data, particularly in regions such as vertebral bodies and arches, the traditional FPS sampling algorithm may struggle to achieve uniform point sampling. In response to this challenge, we implement an enhanced version of the FPS algorithm, integrating distance distribution entropy for improved results. Distance distribution entropy provides a more comprehensive understanding of the distance distribution within the point cloud. By considering the distance distribution of points and their surroundings, it allows for a more intelligent selection of the farthest sampling points, thereby enhancing the informativeness of the sampling process.

For a given point cloud, the first step is to calculate the distances between each point and every other point, forming a distance matrix *D*. The calculation is as follows:

(1) $$H=-\sum_{i}\left(P_{i}log\left(P_{i}+\epsilon \right)\right)$$

In this formula, firstly, the distance matrix *D* is flattened into a one-dimensional array, removing distances on the diagonal (i.e., distances from points to themselves), resulting in an array $D^{\prime }$ containing distances for all pairs of points. Next, the array $D^{\prime }$ is discretized into a histogram to obtain a frequency distribution *P*.

### 3.2. Local Information Extraction Module

The self-attention mechanism, initially proposed for assessing word correlations in different positions, is well-suited for connecting various positions within local point clouds. This adaptability is a significant factor in the success of transformers in handling 3D point clouds.

Once the 3D point cloud is divided into blocks using the KNN algorithm, the self-attention operator is employed to calculate the output features $F_{SA}$ from the corresponding input features $F_{in}$ within a local field *F*. The calculation is as follows:

(2) $$F_{SA}=softmax\left(\frac{QK^{⊤}}{\sqrt{d_{a}}}\right)\left(V\right)$$

where *Q*, *K* and *V* are the query, key, and value metrics, and $d_{a}$ is the dimension of the query and key vectors.

Due to rigid transformations, the absolute coordinates of the same point cloud can vary significantly. To address this, we introduce a position encoding function δ :

(3) δ=ϕ(xyz)

where ϕ is an MLP with two layers and one ReLU nonlinearity.

(4) $$F_{in}=\varphi \left(concat\left(f,\delta \right)\right)$$

where φ is a linear layer, and we do this by concatenating the original features and the transformed position encoding information as the final input features.

In the original model, the Self-Attention (SA) module is enhanced with the Relation Attention (RA) mechanism to improve point cloud feature representation. This is accomplished by using the Laplace matrix *L* (where L=D−A ) to replace the adjacency matrix *A* in the graph convolution network, where *D* is the diagonal matrix. The deviation between the self-attention feature and the input feature is calculated through element-wise subtraction:

(5) $$F_{RA}=F_{in}-F_{SA}$$

where $F_{in}$ is the features of the input, $F_{SA}$ is the features obtained by self attention transformation, and $F_{RA}$ enhances the feature representation of the point cloud.

**Figure 1:**
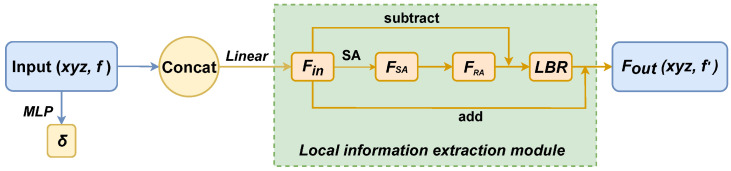
Relative attention (RA), xyz are the coordinates of the input clouds, *f* is the original feature of the input, LBR combines Linear, BatchNorm, and ReLU layers, and SA is the self attention.

Then the characteristics of the final output $F_{out}$ can be expressed as:

(6) $$F_{out}=RA\left(F_{in}\right)=LBR\left(F_{RA}\right)+F_{in}$$

where LBR is a network that combines Linear, BatchNorm and ReLU layers. Figure 1 shows the local information extraction module.

### 3.3. Architecture Design

**Figure 2:**
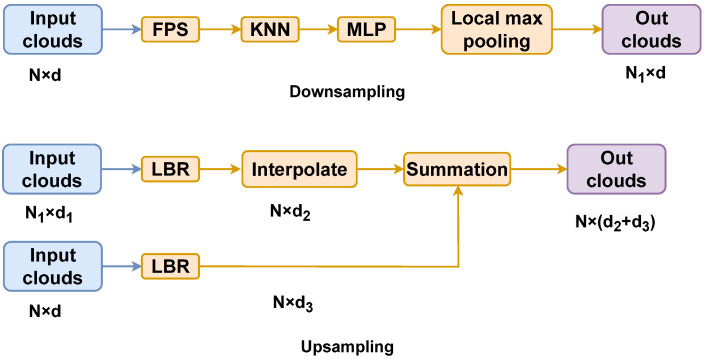
Downsampling and Upsampling. $N_{1}$ and *N* is the number of point clouds, *d*, $d_{1}$ , $d_{2}$ and $d_{3}$ is the corresponding dimension.

The Transformer’s self-attention mechanism excels in point cloud tasks, outperforming PointNet models in benchmarks. Farthest Point Sampling (FPS) ensures comprehensive point cloud coverage and uniformity. In our experiments, FPS sampled points to predict vertebral body upper and lower plate locations using a trained network, reducing the point count. Upsampling for point cloud segmentation follows, as shown in Figure 2.

We adopt the U-Net framework [32], renowned for its excellence in medical image segmentation. For upsampling, we utilize the U-Net’s deconvolution method. After each downsampling, interpolation points preserve previous feature information. Local-global features from skip connections are maintained to ensure invariance. This seamless fusion of local and global features helps mitigate limitations compared to simple concatenation.

Our network begins with input point clouds, expanding dimensionally to 64 through an MLP. It goes through four downsampling stages and four upsampling stages, with the Relation Attention (RA) module applied after each downsampling step.

The input embedding projects the point cloud into a higher-order feature space using an MLP. Downsampling utilizes local max-pooling to capture local-global features, preventing the information loss associated with direct global pooling. Upsampling employs trilinear interpolation, while skip connections aggregate information.

**Figure 3:**
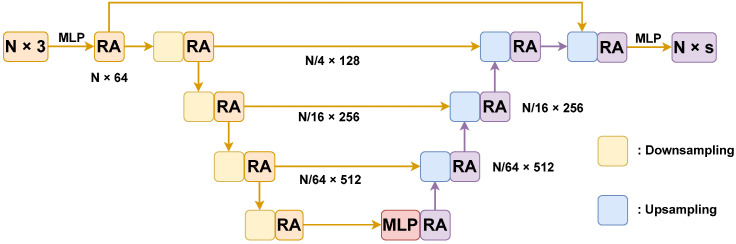
The network. *N* is the number of point clouds input to the network, RA is the local information extraction module, MLP is the multi-layer perceptron, and *s* is the number of point cloud categories.

Our network design, similar to U-Net, incorporates skip connections to enable continuous integration of global and local features, enhancing discriminative feature generation. Figure 3 provides an illustration of our network’s framework.

### 3.4. The Measure of Vertebral Rotation Angle

**Figure 4:**
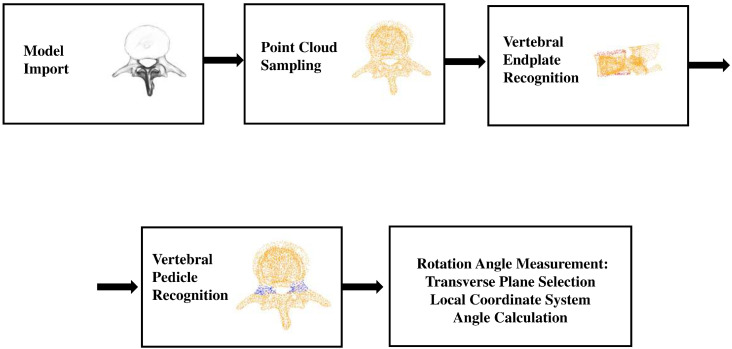
Procedure of the algorithm to calculate the vertebral rotation angle.

The algorithmic steps for the automated measurement of vertebral rotation angles are as follows: First, import the vertebral model and perform point cloud sampling. Next, for the sampled vertebral point cloud, utilize a pre-trained neural network model to predict the corresponding point clouds for the vertebral endplates and pedicle roots. Finally, based on the obtained predicted point clouds, we conduct the measurement of the corresponding point cloud’s rotation angles. Figure 4 shows the procedure of the algorithm to calculate the vertebral rotation angle.

#### 3.4.1. Model Import and Point Cloud Sampling

By utilizing vtk’s three-dimensional reconstruction functionality, we obtained a three-dimensional spine model based on CT images. Subsequently, we employed vtk’s bounding box functionality for manual segmentation, obtaining the corresponding individual vertebra. The respective vertebra models can then be converted into point cloud-formatted data. For the acquired three-dimensional point cloud data, we applied a furthest point sampling algorithm in conjunction with distance distribution entropy, resulting in uniformly distributed point cloud data for the vertebrae.

#### 3.4.2. Vertebral Endplate and Pedicle Recognition

**Figure 5:**
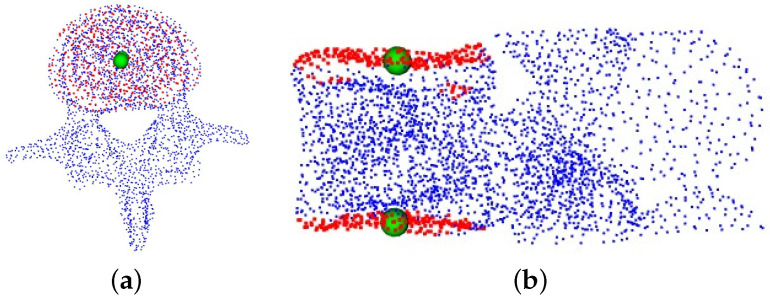
Recognition of vertebral endplate point clouds. (**a**) The top view of the vertebrae; (**b**) the corresponding side view.

We use our network to train on an existing dataset of vertebral endplate point clouds and then apply this trained model to predict new vertebral endplate point clouds. After generating these predicted point clouds, we divide them into two distinct sections based on their spatial distribution. We then determine the centers of these individual segments. Figure 5 displays the expected point clouds for the upper and lower endplates of the vertebrae. The point cloud highlighted in red represents the outcome of our network’s prediction for the vertebral endplates, while the green spheres in the image represent the corresponding endplate centers.

**Figure 6:**
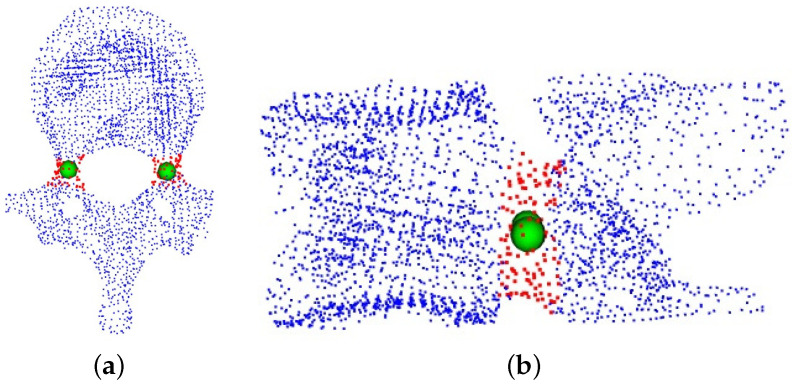
Recognition of the point clouds of vertebral pedicle. (**a**) The top view of the vertebrae; (**b**) the corresponding side view.

We employ our network to train the generated pedicle dataset. For the anticipated pedicle point cloud, we apply the K-means clustering algorithm to partition this particular section of the point cloud into two distinct segments. Subsequently, the corresponding cluster centers are identified. In Figure 6, the highlighted red region represents the point cloud of the vertebral pedicles predicted by our model, while the green spheres symbolize the corresponding pedicle centers obtained through the K-Means clustering algorithm.

#### 3.4.3. Vertebral Rotation Angle Measurement

**Figure 7:**
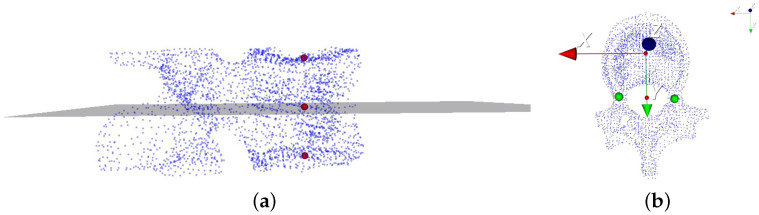
(**a**) The transverse plane of the vertebrae; (**b**) the local coordinate system of the vertebra.

**Figure 8:**
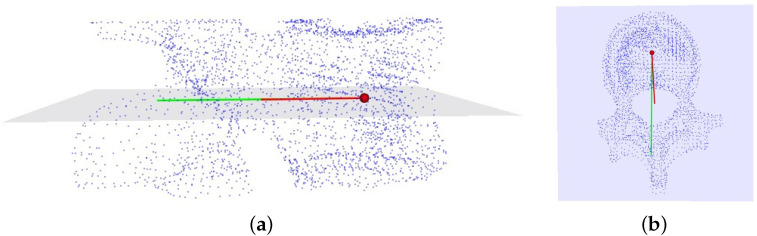
(**a**) The measurement of the vertebral rotation angle; (**b**) the corresponding side views.

To measure the vertebral rotation angle in the 3D vertebral model, we follow a specific methodology:

(1) Transverse Plane Selection: The vertebral centroid, positioned at the midpoint of the line connecting the midpoints of the upper and lower endplates of the vertebrae, serves as the center of the transverse plane. The normal vector of this plane is defined by the line connecting the midpoints of the upper and lower endplates. Figure 7a depicts the vertebra’s transverse plane;
(2) Local Coordinate System: We establish a local coordinate system with the vertebra centroid as the origin point. In this coordinate system, the Y-axis is defined by the line connecting the midpoint of the pedicle and the vertebra centroid. The vector connecting the upper and lower endplates defines the z-axis, while the x-axis is computed using the cross product of the vectors representing the y- and z-axes. Figure 7b illustrates this sequential process;
(3) Angle Calculation: Figure 8 illustrates the method for measuring vertebral rotation angles. This angle is formed between the vector representing the projection of the local coordinate system’s Y-axis onto the global coordinate system’s Y-axis within the vertebra’s transverse plane. Following this methodology, we accurately determine the vertebral rotation angle in the 3D vertebral model. The red and green lines represent the respective projected vectors of the local coordinate system and the global coordinate system’s Y-axis on the transverse plane.

## 4. Experimental Results and Comparisons

In this section, we evaluate the network used in this paper, compare it with other networks, and apply it to the vertebral rotation angle measurement.

### 4.1. Data Preparation

The dataset used in our study is sourced from SpineWeb (dataset3), a freely accessible online resource. Our approach involves downloading the data to facilitate our research endeavors. Each CT image sequence is equipped with element spacing details, accessible directly from the file header. To construct the 3D vertebral model, we harness the power of VTK, an open-source toolkit renowned for its capabilities in three-dimensional reconstruction. This process involves the aggregation of sequential CT images of the vertebrae, enabling us to generate a comprehensive three-dimensional spine model.

Subsequently, we capitalize on VTK’s functionalities to undertake manual segmentation on the model. This intricate segmentation process enables us to precisely isolate and select the specific vertebra of interest from the three-dimensional spine model. The resulting selection serves as the fundamental data source for our analysis, providing a robust foundation for our research efforts.

#### 4.1.1. Generate Point Cloud Data for Vertebral Endplate and Pedicle

**Figure 9:**
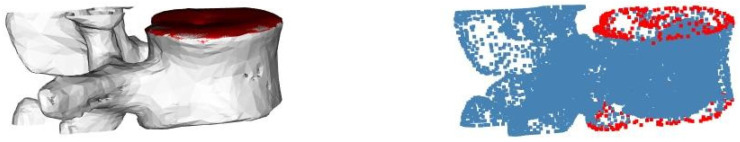
Manual marking of the vertebrae model plane.

Regarding the obtained vertebral model, we perform manual labeling using the marking functionality in vtk. Specifically, we allocate a label of 1 to the point cloud data corresponding to the marked endplates. In contrast, for the remaining point cloud data, we assign a label of 0. The visual depiction of this procedure, illustrating the manual marking of the vertebrae model plane, is presented in Figure 9.

In Figure 9, we provide a comprehensive guide on how to execute the manual labeling process for the upper and lower endplate planes of the vertebrae. This process was facilitated using the versatile vtk open-source toolkit. The left side of Figure 9 illustrates the steps involved in manual labeling using vtk. Following this labeling, we proceed to attribute a label of 1 to the resultant point cloud and designate the label 0 for the remaining point cloud data, as depicted on the right side of Figure 9. This thorough process ensures the accurate distinction of the labeled endplates and sets the stage for subsequent analyses.

**Figure 10:**
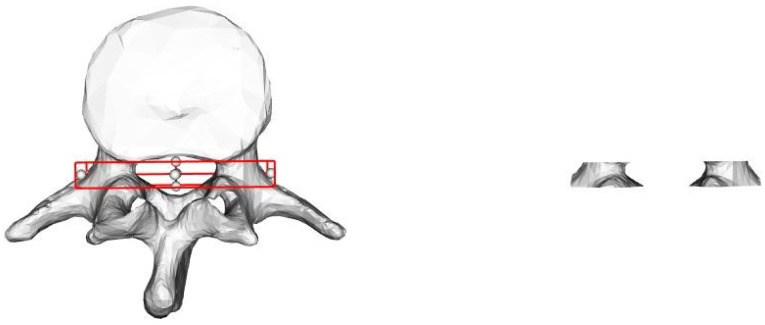
Manual extraction of the pedicle data from the vertebrae model.

Furthermore, we conduct the extraction of data from the vertebral pedicle within the vertebral bone model. This extraction process is primarily facilitated through the utilization of the bounding box feature provided by the VTK toolkit. The operational process of employing the VTK bounding box to effectively capture the vertebrae aligned with the pedicle is graphically depicted in Figure 10.

To elucidate, we designate a label of 1 for the point cloud data that corresponds to the intercepted pedicle of the vertebrae. Conversely, the remaining point cloud data is assigned a label of 0. This comprehensive labeling process not only precisely differentiates the pedicle regions but also sets the groundwork for subsequent analytical pursuits.

#### 4.1.2. Generation Point Clouds and Labels

The training data is generated through the labeling of point clouds corresponding to vertebral endplates, yielding the corresponding training data labels. The presence of multiple point clouds for each vertebral model, coupled with the varying point cloud counts across these models, poses a challenge for directly training subsequent deep learning networks. To address this issue, it becomes necessary to uniformly downsample the point cloud counts to a fixed value, given the varying numbers of point clouds for different vertebral models.

One effective approach for this uniform downsampling is the furthest point sampling algorithm (FPS), which maintains the shape of the sampled point cloud while achieving uniformity in point distribution. By employing FPS, we homogeneously collect labeled point cloud data, transforming it into a structured format comprising 3072 points.

To augment the number of vertebrae within the corresponding labeled dataset, we expand the dataset using two FPS samples drawn from the labeled dataset. This expansion strategy contributes to a more robust and diverse training dataset, thereby enhancing the effectiveness of subsequent deep learning processes.

### 4.2. Evaluating the Network

First, we evaluate the network using the publicly accessible ShapeNet dataset and conduct comparisons with other networks. The ShapeNet dataset has 16 categories and a total of 16,881 shapes annotated with 50 parts. The Shapenet dataset comprises 3D models from multiple categories, encompassing objects of various shapes and types. This allows for an examination of model performance across diverse object types in comparative studies of segmentation networks. Furthermore, it is widely used as a standard dataset in point cloud research.

We train using the Adam optimizer with a learning rate of 0.003, a decay rate of 0.5, a batch size of 4, and an epoch of 200.

During the training process, we utilize the cross-entropy loss function. We compare the obtained probability distribution from the Softmax function with the true labels and compute the cross-entropy loss. If the true labels are represented as a one-hot encoded vector *y*, where the i-th element $y_{i}$ indicates whether the sample belongs to the i-th class, the cross-entropy loss is calculated as:

(7) $$L\left(y,softmax\left(z\right)\right)=-\sum_{i=1}^{C}y_{i}log\left(softmax{\left(z\right)}_{i}\right)$$

where the raw output *z* is a C-dimensional vector, and *C* is the number of component categories in the point cloud.

IoU represents, in point cloud segmentation, the ratio of the intersection and sum of the true labels and predicted values for the class. mIoU is the mean intersection over union for each class in the dataset.

(8) $$mIoU=\frac{1}{K}*\sum_{k=1}^{K}\frac{TP_{k}}{TP_{k}+FP_{k}+FN_{k}}*100\%$$

where *K* represents the number of categories, $TP_{k}$ represents the number of correctly predicted point clouds in category *k*, $FP_{k}$ represents the number of point clouds that were misclassified as category *k*, and $FN_{k}$ represents the number of point clouds where points of category *k* are misclassified as other categories.

We evaluate our segmentation results on the ShapeNet dataset using the mean intersection over union (mIoU) as our evaluation metric. Table 1 presents these results.

Table 1 reveals that our network’s performance does not consistently surpass other point cloud segmentation networks across all ShapeNet dataset categories. However, our method excels in specific categories, particularly those featuring complex structures and smaller, indistinct components. Notably, our network demonstrates exceptional segmentation accuracy when dealing with objects characterized by complex structures, such as the Motor category. Furthermore, due to the complex shape of vertebrae, our network is well-suited for segmenting vertebral data.

Table 2 illustrates whether the combination of distance distribution entropy with FPS influences the accuracy of predicting the vertebral dataset. From Table 2, it is evident that the results obtained after preprocessing vertebral point cloud data with distance distribution entropy are significantly superior to those obtained without using distance distribution entropy.

### 4.3. Pedicle Recognition Effect

Accurate angle measurement relies on establishing the Y-axis of the local coordinate system through two pedicle centers. Precise pedicle recognition is critical for minimizing potential errors, making the statistical analysis of pedicle recognition essential.

Table 3 presents a randomly selected subset of 10 vertebral model datasets from the complete dataset. Given the intricate spatial structure of the vertebrae, our network demonstrates superior segmentation accuracy compared to alternative networks in the majority of vertebral datasets.

**Figure 11:**
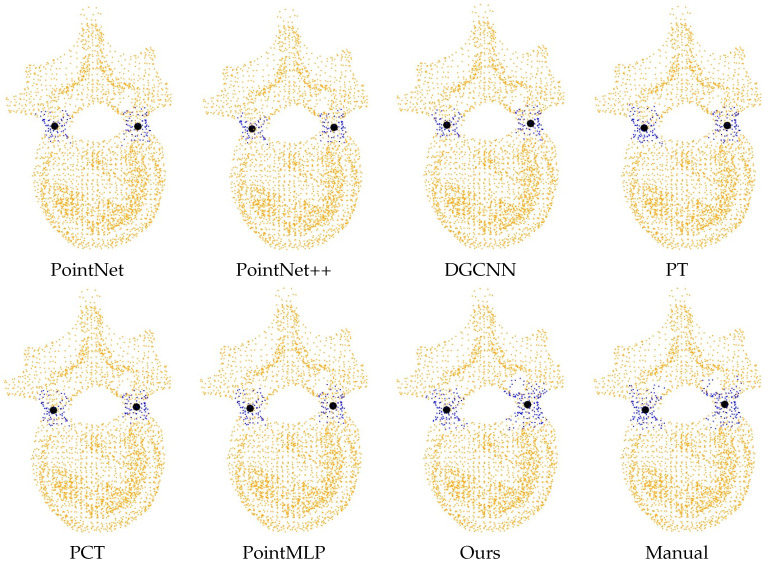
Demonstration of the clustering effect of the pedicle point clouds segmented in different ways.

Figure 11 vividly illustrates the clustering results for segmented vertebral body point clouds using different techniques. The blue point cloud in the figure represents the predicted vertebral pedicle point cloud, and the corresponding black spheres depict the clustering centers obtained through K-means clustering. By comparing it with the manually segmented vertebral pedicle point cloud, it can be observed that our proposed method outperforms other networks in predicting vertebral pedicle point clouds (the predicted blue point cloud is closer to the manually segmented point cloud). Our experiments reveal that K-means effectively groups various methods for obtaining vertebral pedicle point clouds into two clusters, closely aligned with actual pedicle centers. Notably, our method consistently produces clustering centers that closely match manual segmentation, emphasizing its effectiveness and accuracy in this critical analysis.

We conduct experiments on 10 randomly chosen vertebral bone datasets, calculating the displacement distances between their clustering centers and those obtained from manual segmentation. Table 4 displays the average offset distance. We use the distance (mm) as the evaluation metric. Our proposed network generates centroids that closely match manually derived centroids when using the K-means clustering algorithm. These segmentation results underscore the potential of our network in establishing the local coordinate system of vertebrae and computing vertebral rotation angles. This comprehensive assessment demonstrates the effectiveness and promise of our approach for improving vertebral rotation angle measurements, with potential applications in clinical evaluation and diagnosis.

### 4.4. Measurement Results

We initially select 10 vertebral models randomly and then conduct measurements using both manual and automated methods. For manual measurements, 3 observers measure each vertebra 10 times, and the results are averaged. Additionally, we employ our automated measurement program to measure each vertebra 10 times.

Manual measurements involve marking the four points of the vertebral endplate (top, bottom, left, and right) and determining the midpoint of these points to establish the endplate’s center. We then extract the corresponding point cloud of the pedicle using an envelope box. The K-means algorithm is utilized to find the cluster centers of the left and right pedicles, and the corresponding angles are calculated.

**Figure 12:**
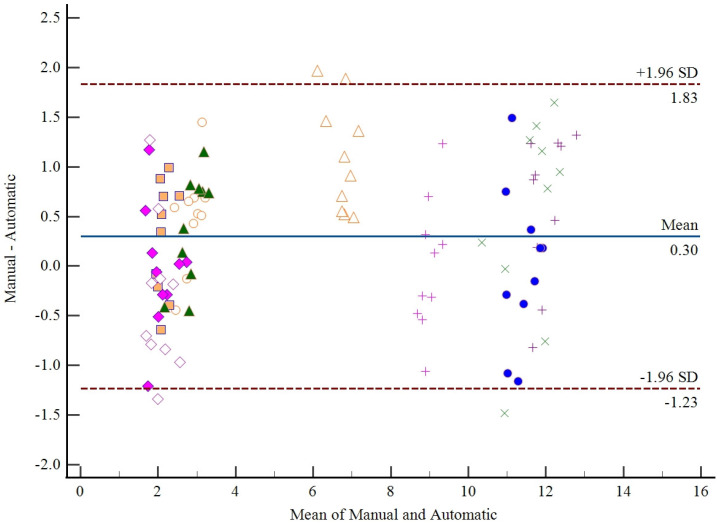
Bland–Altman plots (i.e., the difference between two measurements plotted against their mean) for measurements from manual and automatic measurements. Additional horizontal lines correspond to the mean difference of the measurements (dashed-dotted) and to the lines of agreement (dashed), i.e., the 95% CI for the difference of the measurements. The 10 different patterns in the figure represent 10 different vertebral models.

To validate our proposed automated measurement method, we randomly selecte10 vertebral models and conduct both manual and automated measurements, each repeated 10 times. Figure 12 visualizes the measurement variability using Bland–Altman plots, which display a total of 100 points distributed across 10 distinct patterns. Each point represents a measurement pair, and points within the same pattern correspond to the same study subject. Importantly, points within the same pattern cluster closely together, indicating consistency within each subject. It is noteworthy that only 4 points (4% of the data) fall outside the limits of agreement (LoA).

This outcome suggests that there is no significant systematic difference when comparing differences between manual and automated measurements. The majority of measurements closely align, affirming the validity of our proposed automated measurement method.

Table 5 shows the final results of the manual measurements as well as the average of the automatic measurements. The table presents the mean of 10 randomly selected vertebral models subjected to 10 manual measurements by 3 observers and the mean of 10 automated measurements. The analysis of the error in the automatic measurement of the angle is mainly due to the fact that the center of the upper and lower endplate point cloud of the predicted vertebrae is used in the generation of the vertebral medial surface, which may lead to deviations in the final angle measurement due to incomplete segmentation of the vertebral endplate point cloud. To address this issue we took multiple automated measurements to average the results of their automated measurements.

To evaluate the accuracy of the automatic angle measurement, we use the evaluation metric ICC, and the model we use is a two-way mixed model of multiple measurements and absolute agreement (Table 6). Its calculation formula is as follows:

(9) $$ICC=\frac{MS_{R}-MS_{E}}{MS_{R}+\frac{MS_{C}-MS_{E}}{n}}$$

where $MS_{R}$ represents the mean square for rows, $MS_{E}$ represents the mean square for error, $MS_{C}$ represents the mean square for columns, and *n* represents the number of subjects.

The mean differences and the 95% confidence intervals between the manual measurements were: $0.56^{\circ }\pm 0.40^{\circ }$ , $-0.39^{\circ }\pm 0.54^{\circ }$ , $0.33^{\circ }\pm 0.63^{\circ }$ (Observe1-Observe2, Observe1-Observe3, Observe2-Observe3). The results between automatic and manual measurements were: $-0.47^{\circ }\pm 0.47^{\circ }$ , $-0.42^{\circ }\pm 0.47^{\circ }$ , $-0.09^{\circ }\pm 0.43^{\circ }$ (Automatic-Observe1, Automatic-Observe2, Automatic-Observe3). The difference between automatic measurement and manual measurement is at the same level as the difference between manual measurements performed by different observers.

Using the intraclass correlation coefficients in Table 6, we can see that the ICC ranges between 0.980 and 0.993 , which indicates that there is a high level of consistency between manual measurements and the automated measurements proposed by us.

### 4.5. Performance and Results Analysis

Comparative evaluations between our automated measurement method and manual measurements reveal a high level of agreement, supported by the Bland–Altman plots and ICC values. The strong agreement observed between automatic and manual measurements, as well as among different manual observers, suggests that our automated method has the potential to effectively replace manual measurements. However, Table 6 indicates a slight decrease in ICC values between automatic and manual measurements and among different manual measurements. This decrease is attributed to the estimated offset of the vertebral body center and the corresponding offset of the pedicle center.

Our local information extraction module, incorporating location codes into original input features and computing local features using our proposed relation attention mechanism, plays a pivotal role in our approach. The experimental results highlight our network’s superiority in segmenting specific object parts, particularly excelling in segmenting vertebral point cloud data, including the upper and lower endplates of vertebrae and partial point clouds of the pedicle. Comparative analyses with other networks consistently demonstrate our network’s superior performance in both endplate and pedicle segmentation.

However, our experiment has some limitations, one of which stems from the computational load introduced by incorporating a local point cloud information extraction module in our network. While this addition results in a certain improvement in training accuracy, it also increases the computational workload during the training process, thereby impacting the operational efficiency of the network. Therefore, in the future, we plan to explore modifications to this local information extraction strategy to enhance the operational efficiency of the network.

Another limitation stems from our approach to establish the symmetry plane of vertebrae using centroids based on upper and lower endplate point clouds. This approach may encounter challenges with incomplete segmentation of the vertebrae point cloud, such as partial endplate segmentation. In cases of uneven segmentation, our method, while improving endplate recognition to some extent, might produce incorrect centroid predictions due to non-uniform segmentation.

To address this limitation, future efforts should aim to enhance the vertebrae point cloud segmentation network by incorporating inherent features of vertebrae point clouds. This customized approach could lead to more accurate segmentation results, effectively addressing the unique characteristics of vertebrae models. Additionally, modifying the algorithm to incorporate insights from point cloud completion and other techniques could improve measurement accuracy for incomplete vertebrae point cloud models. These refinements will contribute to a more robust and comprehensive measurement methodology suitable for complex real-world scenarios in clinical applications.

## 5. Conclusions

In this paper, we successfully introduce an automated measurement method based on three-dimensional vertebral models, particularly suitable for evaluating spinal deformities in idiopathic scoliosis patients. Compared to traditional manual measurement methods, our automated approach demonstrates significant advantages in terms of accuracy and efficiency. Through comparisons with manual measurements, we validate the consistency of our automated measurement method among different observers and showcase its superior performance in vertebral model segmentation. Despite the significant success of our method, we acknowledge some limitations in the experiment. In future research, we focus on improving the local information extraction strategy to enhance the operational efficiency of the network. Additionally, efforts are directed towards further optimizing the vertebrae point cloud segmentation network to overcome challenges associated with incomplete segmentation. We believe these improvements will facilitate the application of our method in complex clinical scenarios, providing a more accurate and reliable tool for the assessment of spinal deformities.

## Figures and Tables

**Table 1:** The result for the part segmentation on the ShapeNet. The metric is mIoU(%) on points.

	Mean	Airplane	Bag	Cap	Car	Chair	Earphone	Guitar	Knife	Lamp	Laptop	Motor	Mug	Pistol	Rocket	Skate Board	Table
PointNet	81.5	79.8	65.6	75.4	68.9	87.9	68.3	88.8	82.2	77.6	94.8	44.2	85.8	75.0	50.7	70.6	81.5
PointNet++	83.6	81.4	75.1	81.0	***76.7***	89.8	***78.2***	90.1	81.5	82.3	95.2	60.8	90.6	78.5	52.9	72.5	81.2
DGCNN	83.9	80.9	65.1	68.4	75.7	***90.3***	69.5	89.8	84.9	***84.5***	95.3	48.3	87.9	74.3	42.9	70.6	***82.9***
Point Transformer	81.4	78.3	74.3	78.4	69.8	88.9	75.4	89.4	83.5	81.7	94.7	60.0	80.2	74.6	49.9	68.5	78.4
Point Cloud Transformer	83.2	***82.0***	67.7	***82.2***	73.0	89.2	76.0	89.6	85.0	80.6	95.2	51.9	89.1	78.1	49.1	69.6	82.1
PointMLP	84.3	81.2	70.7	80.4	76.2	90.1	73.2	90.1	86.6	83.1	***95.5***	56.4	91.1	***80.7***	48.3	***74.6***	82.8
Ours	83.6	79.1	***75.2***	78.6	74.4	89.4	74.6	***90.2***	***86.7***	83.4	95.0	***61.1***	***91.4***	78.5	***54.6***	68.8	82.4

The bold and italicized font indicates the best segmentation performance compared to other networks for that category.

**Table 2:** The result for the part segmentation on the vertebral dataset. The metric is mIoU(%) on points.

	1	2	3	4	5	6	7	8	9	10
FPS	95.1	95.9	96.5	93.7	96.1	97.0	98.1	96.3	97.3	97.1
FPS + entropy	95.3	96.3	97.1	96.1	96.4	96.6	98.6.5	96.5	97.6	97.3

**Table 3:** The result for the part segmentation on the vertebral dataset. The metric is mIoU(%) on points.

	1	2	3	4	5	6	7	8	9	10
PointNet	93.7	96.1	96.9	91.5	***97.6***	95.8	94.6	95.4	97.6	96.4
PointNet++	94.7	95.1	96.3	94.5	96.9	96.1	97.5	95.1	97.2	97.5
DGCNN	95.4	96.1	96.3	97.7	97.2	97.1	98.7	96.4	97.4	97.5
Point Transformer	95.3	95.4	97.1	93.4	96.8	96.4	97.2	96.3	97.3	96.7
Point Cloud Transformer	94.6	95.3	96.3	94.8	97.2	95.5	94.6	94.6	97.4	97.2
PointMLP	92.6	95.7	97.1	***98.3***	97.0	97.2	97.3	96.1	97.2	97.2
Ours	***95.6***	***96.2***	***97.3***	94.9	97.2	***97.2***	***98.9***	***96.7***	***97.8***	***97.6***

The bold and italicized numbers indicate the best segmentation performance for the corresponding vertebra.

**Table 4:** Comparison of the average offset distances between the clustering centers of multiple network-based pedicle segmentation results on the test set with respect to the reference cluster centers derived from manually labeled pedicles. We use the distance (mm) as the evaluation metric.

	PointNet	PointNet++	DGCNN	PT	PCT	PointMLP	Ours
Left Pedicle	3.621	1.823	3.947	2.479	1.906	2.075	1.773
Right Pedicle	2.738	1.960	4.520	2.059	2.007	2.560	1.188

**Table 5:** Manual and automatic measurement results. The metric is °.

Model	Observe1	Observe2	Observe3	Automatic
1	3.57	2.69	3.11	2.62
2	12.85	12.33	12.12	11.70
3	11.31	10.85	11.87	11.40
4	12.38	12.78	10.53	11.35
5	8.95	8.81	9.23	8.99
6	7.44	7.47	7.08	6.20
7	2.44	2.64	1.92	2.01
8	2.46	3.57	3.20	2.67
9	2.20	2.30	1.67	2.09
10	2.34	1.94	1.36	2.19

**Table 6:** ICC using a two-way mixed model with absolute agreement, comparing the correlation in-between manual measurements and automatic measurements.

	ICC	95% CI
Automatic-Observe1	0.987	[0.904, 0.997]
Automatic-Observe2	0.985	[0.929, 0.996]
Automatic-Observe3	0.991	[0.967, 0.998]
Observe1-Observe2	0.993	[0.971, 0.998]
Observe1-Observe3	0.983	[0.933, 0.996]
Observe2-Observe3	0.980	[0.925, 0.995]

## Data Availability

Data is contained within the article.
